# Orange juice as dietary source of antioxidants for patients with hepatitis C under antiviral therapy

**DOI:** 10.1080/16546628.2017.1296675

**Published:** 2017-03-22

**Authors:** Danielle Gonçalves, Claudia Lima, Paula Ferreira, Paulo Costa, Angela Costa, Walter Figueiredo, Thais Cesar

**Affiliations:** ^a^ Food and Nutrition Department, School of Pharmaceutical Sciences, Universidade Estadual Paulista (UNESP), Araraquara, Brazil; ^b^ Clinical Analysis Department, School of Pharmaceutical Sciences, Universidade Estadual Paulista (UNESP), Araraquara, Brazil; ^c^ Special Health Service of Araraquara (SESA), Faculty of Public Health, Universidade de Sao Paulo (USP), Araraquara, Brazil

**Keywords:** Orange juice, chronic hepatitis C, biochemical markers, oxidative stress, nutritional status, antioxidants

## Abstract

**Background:** HCV causes alterations in liver metabolism, resulting in biochemical and nutritional disorders. Supplementation with antioxidants has been suggested to minimize the diseases effects.

**Objective:** This study assessed whether orange juice, a source of citrus flavonoids and vitamin C, may contribute to the treatment of patients with chronic hepatitis C.

**Design:** Anthropometric, hemodynamic, dietary, and biochemical parameters, CRP and liver enzymes were measured in 43 adult patients of both genders who were diagnosed with chronic hepatitis C and were under antiviral therapy. Twenty-three patients were supplemented with orange juice for eight consecutive weeks, while 20 were enrolled as control group.

**Results:** Following regular use of orange juice, no alterations were found in body mass, fat, and waist circumference. The serum levels of total cholesterol, LDL-cholesterol, CRP and parameters of oxidative stress decreased in the orange juice group. Furthermore, the levels of the liver enzyme AST decreased in those who had high levels before the intervention.

**Conclusion:** The orange juice was a convenient food in the diet of patients due to the increase in antioxidant capacity and decreased inflammation and cholesterol in blood serum, in addition to maintaining body mass, which protect against the harmful effects caused by the chronic hepatitis C virus.​​​

## Introduction

Hepatitis C is characterized by liver inflammation resulting from infection by hepatitis C virus (HCV), which over time can lead to complications such as liver fibrosis, cirrhosis and hepatocellular carcinoma. The presence of HCV in the cells results in oxidative stress due to the increase of cell metabolism, with the reduction of antioxidant enzymes and the increase of liver enzymes alanine transaminase (ALT) and aspartate transaminase (AST).[1] HCV carriers exhibit high levels of proinflammatory cytokines, such as tumor necrosis factor alpha (TNF-α), which inhibit insulin signaling in the hepatocytes, and thus increase the risk of insulin resistance and hepatic steatosis.[2]

Patients with chronic hepatitis C also exhibit high serum levels of C reactive protein (CRP), which is a marker of inflammation. A correlation has been suggested between increased CRP levels and lipid metabolism disorders in patients with chronic hepatitis C.[3] Several studies have shown that HCV follows the lipoprotein pathway inside the hepatocytes to survive in the host and increase its viral load. Subsequent to the entry of HCV into cells, the virus replicates in the endoplasmic reticulum, and is secreted into the bloodstream bound to VLDL.[4] Under such conditions HVC exhibits a high degree of infection; conversely, the free virus infection effectiveness is low.[5]

HCV is a positive-sense single-stranded RNA virus (Flaviviridae family) that contains 9600 nucleotides and, because of its variability, it is classified into six major genotypes designated 1 to 6 according to the sequence in the primary structure, with three major subtypes: a, b and c. The choice and monitoring of antiviral therapy is based on the HCV genotyping test of the patients, which aims to reduce viral replication and delay disease progression.[6] However, this therapy is associated with side effects, including nausea and anemia, which cause nutritional depletion and increase oxidative stress. Some studies have consistently suggested that antioxidant supplements might minimize the deleterious effects of both the virus and pharmacological treatment.[7]

Freshly squeezed or pasteurized orange juice includes nutrients and bioactive compounds with therapeutic properties, including vitamin C, carotenoids, and citrus flavanones.[8] A portion of​​​ 500 ml of orange juice made from sweet oranges (*Citrus sinensis*) contains around 168 mg of vitamin C, 3.65 mg of carotenoids, 16.4 mg of hesperidin​​​ and 2.7 mg of naringin,[9] which exhibit antioxidant, hypolipidemic, and anti-inflammatory properties.[10] Those flavonoids reduce the 3-hydroxy-3-methyglutaryl coenzyme A reductase (HMG-CoA reductase), acetyl-Coenzyme A acetyltransferase 2 (ACAT2) and microsomal triglyceride transfer protein (MTP), which control the hepatic synthesis of cholesterol and triglycerides.[11] *In vitro* studies point to the efficacy of naringenin against HCV production by reducing or inhibiting these enzymes activity.[12]

Although previous studies suggest that antioxidants might improve oxidative imbalance caused by HCV infection,[13,14] there are no studies on the effect of citrus juice on infected patients. In addition, HCV infected patients were chosen instead of HBV and HAV, because of its higher prevalence (> 60% worldwide), and although the other types of hepatitis are more lethal, they are acute episodes and can be prevented​​​ by vaccination. Thus, the objective of this study was to show that orange juice can play a supporting role in the treatment of chronic hepatitis C. For that purpose, the consumption of orange juice was tested in patients with chronic hepatitis C and treated with antiviral therapy, and the nutritional status and biochemical and oxidative stress parameters were assessed and compared to the control group.

## Material and methods

### Subjects

This was a randomized clinical trial, controlled, parallel-arm study, conducted in individuals with chronic hepatitis C. Uninfected subjects were not included in this proposal because the effect of an antioxidant supplement would not be perceived in such condition. Screening of volunteers was performed at Special Health Service of Araraquara (SESA), which is a region reference center for infectious diseases. Co-authors MD Figueiredo and NP Costa assisted the screening of patients with chronic hepatitis C, and MS Gonçalves and MS Lima performed the interviews. During the interview, HCV patients were investigated with regard to comorbidities, use of drugs, consumption of alcohol and smoking. All volunteers were invited to participate in the study: patients registered at SESA, aged ⩾18 years, positive HCV-RNA serum and under hepatitis C treatment. Volunteers were evaluated for viral co-infection before the beginning of this study, using immunological tests recommended by the Brazilian Ministry of Health: HBsAg, total anti-HBc for hepatitis B; anti-HAV total and IgG for hepatitis A; anti-HIV 1/2 for HIV. The inclusion criteria of the subjects were the detection of circulating HCV RNA by polymerase chain reaction using the COBAS AMPLICORTM HCV 2.0 assay (Roche, Branchburg, NJ, USA) and negative HBV surface antigen or antibodies to HIV. Viral genotyping was performed using the HCV Genotype 2.0 Assay-LiPA (Versant®HCV-Siemens, Tarrytown, NY, USA). The exclusion criteria were co-infection with other viruses as hepatitis B virus (HBV) and human immunodeficiency virus (HIV), and presence of diabetes mellitus or ascites, and elevation in serum ferritin levels.

Seventy patients with chronic hepatitis C, who had undergone liver biopsy before treatment with drugs, were enrolled. The histological classification of liver biopsy was according to the METAVIR scale (Brazilian Ministry of Health), where F0 represents a normal liver architecture without fibrosis; F1 shows portal fibrosis without septa; F2 indicates portal fibrosis with rare septa; F3 shows numerous septa without fibrosis and, F4 indicates cirrhosis. All patients were being treated with pegylated interferon combined with ribavirin, according to the Clinical Protocol and Therapeutic Guidelines for Viral Hepatitis C and Co-infections of the Brazilian Health Ministry.[15] This study followed the guidelines of the Declaration of Helsinki and all patients signed an informed written consent. The Ethical Board on Research of the School of Pharmaceutical Sciences of UNESP approved all procedures involving human patients (Protocol #46/2011). This clinical study has been declared to the website ClinicalTrials.com under the number NCT03026569.

### Experimental protocol

The subjects were divided into two groups: (1) control, with no regular consumption of orange juice; and (2) orange juice group, supplemented with orange juice (500 ml day^–1^) for eight weeks. Each participant of the second group was given 4 l week^–1^ of 100% commercial pasteurized orange juice (Pera Rio variety) produced by Citrosuco, Matao, SP, Brazil®. They were instructed to drink orange juice in two daily portions over the eight consecutive weeks of the experiment. Patients from both groups were asked to maintain their usual lifestyle, diet, and physical activity, and they were checked weekly by the researcher’s team. Assessment of anthropometric data, dietary intake, as well as blood sample collection for biochemical analysis, was performed in all participants on the first and last day of the experiment.

### Assessment of anthropometric data

Body mass was measured by trained personnel using a digital scale, and the height was measured using a standing stadiometer. Waist circumference was measured midway between the costal margin and iliac crest. Biceps, triceps, subscapular, and suprailiac skinfolds were measured using a Lange Skinfold Caliper®, and the patients’ total body fat was calculated.[16] All measures were made three times and the average was calculated.

### Assessment of food and nutrient intake

Dietary intake assessments included three 24-h dietary recalls, collected in two weekdays and one weekend day, and one food-frequency questionnaire. The study dietitians made two complete dietary evaluations, in the beginning and in the end of the experimental period. The participants were previously instructed about food portions to facilitate estimation of intake. Daily energy and nutrient intakes were estimated using the software ‘NutWin’ version 3.1, Sao Paulo School of Medicine – Federal University of São Paulo (UNIFESP).

### Biochemical assessment

A blood sample (32 ml) was collected for biochemical assessment following a 12 h fast at the beginning and the end of the experimental period. The blood serum was separated by centrifugation at 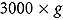 for 10 min and stored at −80°C until the analyses performance. The tests were performed at the Diagnostic Reference Center Laboratory, FCFAR-UNESP. The serum concentrations of total cholesterol, HDL-cholesterol, LDL-cholesterol, triglycerides, glucose, CRP, AST, ALT, gamma-glutamyl transpeptidase (γ-GT), and alkaline phosphatase (ALP) were assessed using Labtest® kits (Lagoa Santa, MG, Brazil) in a photometric system using the Labmax 240® device (Labtest®, Lagoa Santa, MG, Brazil). Insulin was assessed by electrochemiluminescence, and LDL-cholesterol was calculated according to Friedewald et al. (1972) [17]. ​​​Insulin resistance was calculated using the homeostasis model assessment index (HOMA-IR),[18] and >2.71 was used as the cutoff point for the Brazilian population.

#### Oxidative stress assessment

​​​The 2,2´-azinobis(3-ethylbenzthiazoline sulfonate) (ABTS) [19] assay was used to determine the total antioxidant capacity of subjects’ serum. In this method, an ABTS^•+^ radical is generated in the assay and the antioxidant activity of the sample against the radical is measured according to the reduction of the ABTS^•+^ by the hydrogen-donating antioxidant present on the sample. Trolox was used as standard. Five µl of a 7 mM solution of ABTS was added along with 88 ml of a 140 mM potassium persulfate solution and the mixture left at room temperature, in the dark for 16 h. Before use, the solution was diluted (1:88) with a 10 mM sodium phosphate buffer, pH 7.4 (initial absorbance at 734 nm of 0.7). Five μl of Trolox standard (0, 0.50, 0.75, 1.00, 1.25, 1.50, 1.75, 2.00 mM) and 5 μl of each serum samples were mixed with 300 μl of ABTS^+^ solution. After 6 min, absorbance at 734 nm was measured in a microplate reader (Epoch, Biotek, Winooski, VT, USA). ​​​Total antioxidant capacity was based on the molar extinction coefficient of Trolox obtained by an analytical curve.

The thiobarbituric acid-reactive substances (TBARS) assay was used as an indicator of lipid peroxidation in subject serum.[20, Although not specific for lipid peroxides, because thiobarbituric acid (TBA) also reacts with protein, sucrose and metabolites in the plasma, it is a usual method for this purpose. For the assay performance the 1,1,3,3-tetraethoxypropane (TEP) was used as standard for malondialdehyde (MDA) equivalents (1 mol TEP = 1 mol MDA in reacting with thiobarbituric acid – TBA). Two hundred μl of MDA standard (0, 1.25, 1.88, 2.50, 3.13, 3.75, 6.25, and 12.50 μM) and 200 μl of each serum sample were mixed with 200 μl of sodium dodecyl sulfate (SDS) and then 500 μl of staining reagent (5.3 mg ml^–1^ of TBA diluted in 20% acetic acid, pH 3.5) were vortexed, incubated at 100°C for 60 min, and cooled on ice for 10 min. The standards and samples were centrifuged at 8000 × g for 10 min, and the absorbance of the supernatant was determined at 532 nm in a microplate reader (Epoch, Biotek, Winooski, VT, USA). TBARS concentration was based on the molar extinction coefficient of MDA obtained by an analytical curve.

### Statistical analysis

All parameters before and after treatment were compared between the juice group and the control group using the general linear model of repeated measures analysis. Differences in baseline patients’ characteristics between the two groups were analyzed by one-way analysis of variance (ANOVA). The LSD signed rank post-test was used to assess significant changes in parameters after the start of treatment in each group. All data are expressed as means ± S.D. Differences were considered statistically significant at *p* < 0.05. The analyses were performed by software IBM SPSS Statistics (v.21, SPSS: an IBM Company, Chicago, IL, USA).

## Results

### Study population

From 70 screened patients, 22 subjects did not meet eligibility criteria, and 48 were random divided into the orange juice (*n* = 24) and control group (*n* = 24). None of them was on lipid-lowering drugs that could be a confounder effect, since the drug blocks replication and release of HCV. During the course of the experiment, one patient was excluded orange juice group due to high levels of ferritin, and four were excluded from control group due to diarrhea (one) and to no adherence to hepatitis drugs (three). At the end, 43 participants consistently adhered to the experimental protocol, as control group (*n* = 20) and orange juice group (*n* = 23). Patients from the orange juice group reported high compliance to the juice supplementation.

Clinical records of the patients showed a predominance of chronic infection by HCV genotype 1 (69.4%), followed by genotypes 3 (25.8%) and 2 (4.8%). Furthermore, liver biopsy showed that the majority of patients (68%) had moderate inflammatory activity, ranging from F0 grade without fibrosis (8%); F1 grade with portal fibrosis (36%); F2 grade with scars in the liver areas that contains blood vessels (16%); F3 grade with bridges of fibrosis connected to other areas with fibrosis (28%); and finally, F4 grade with advanced scars or more severe fibrosis with cirrhosis (12%).[15]

### Assessment of anthropometric data

Anthropometric data before and after the period of intervention with orange juice are presented in Table 1. No differences were detected in body weight, BMI, body fat and waist measures of the participants in the beginning or in the end of the study, for both groups (orange juice and control).

**Table 1.** Anthropometrical and dietary intake from chronic hepatitis C patients before and after supplementation of orange juice.

	Orange juice (*n* = 23)		Control (*n* = 20)	
Parameters	Baseline	After	Baseline	After
***Anthropometrical***				
Body mass (kg)	72.6 ± 12.8	71.6 ± 12.1	71.9 ± 11.5	72.0 ± 11.8
BMI (kg m^–^^2^)	23.2 ± 3.8	22.8 ± 3.5	24.6 ± 3.6	24.6 ± 3.6
Waist circumference (cm)	91 ± 13	90 ± 13	89 ± 10	90 ± 10
Body fat (%)	35 ± 12	33 ± 10	28 ± 7	29 ± 7
***Dietary intakes***				
Energy (kcal)	2080 ± 623	2146 ± 531	2348 ± 978	2403 ± 953
Carbohydrates (g)	252 ± 68	283 ± 66	312 ± 137	320 ± 133
Lipids (g)	86 ± 35	77 ± 24	85 ± 46	85 ± 46
Protein (g)	72 ± 23^a^	87 ± 25^b^	104 ± 40^b^	104 ± 41^b^
Calcium (mg)	583 ± 264	544 ± 241	768 ± 381	760 ± 359
Iron (mg)	11.6 ± 3.9^a^	13.3 ± 4.2^b^	14.7 ± 5.5^b^	14.8 ± 5.6^b^
Vitamin C (mg)	80 ± 53^a^	247 ± 72^b^	126 ± 181^a^	124 ± 181^a^
Folate (μg)	131 ± 58^a^	174 ± 38^b^	97 ± 77^a^	96 ± 79^a^
Vitamin E (mg)	37 ± 20	34 ± 10	22 ± 16	23 ± 16
Sodium (g)	2.3 ± 0.6	2.1 ± 0.7	3.1 ± 1.3	3.0 ± 1.3


BMI, body mass index.

Mean values ​​± SD before and after ingestion of 500 ml day^–1^ orange juice for eight weeks.

General linear model of repeated measures analysis followed by one-way and post-hoc LSD test, *p* ≤ 0.05.

Values with the same letter in a row are not significantly different, while the different letters are statistically significant.

### Assessment of food and nutrient intake

Total energy, carbohydrate, lipid, protein, vitamins and minerals were obtained from three-day dietary records at the beginning and end of the experiment, and the results are presented in Table 1. The two groups did not differ significantly for total energy consumption, macronutrients and micronutrients, except for protein and ion intake, which were higher in the control group before the study. After intervention with orange juice, increases of protein (21%) and iron (15%) were detected in this group, which became similar to the control group. As expected, intakes of vitamin C (209%) and folate (33%) were higher in the juice group. No changes were observed in the dietary intake for control group. A summary of the food items consumed by both groups, separated in food groups, is showed on Table 2. No difference was found between the orange juice group and the control group, demonstrating very similar choices of foods and food groups for all subjects.

**Table 2.** Main food items often consumed by all patients separated in food groups.

Food group	Daily consumption	Weekly consumption
Cereal	Bread, rice, sweet cookies, cream cracker	Potato, pasta, cake, cassava
Vegetable	Tomato, lettuce, spinach, zucchini, cabbage, chayote	Carrot, eggplant, broccolis, arugula
Fruits	Banana, apple, papaya	Pear, peach, mango, orange
Protein	Beans, meat, poultry, pork	Green beans, eggs, nuts, ham, sausage
Milk	Milk, yogurt	Cream cheese, mozzarella
Fats, oils and sweets	Margarine, butter, chocolate, candies	Sweet desserts (jelly fruit)
Drinks	Coffee, sugary beverages, fruit juice	Sodas, tea

### Biochemical assessment

In the post-intervention assessment, the serum concentrations of total cholesterol and LDL-cholesterol decreased only in the juice group by 9.5% and 12.5%, respectively.(Table 3) No changes were found in the HDL-cholesterol and triglycerides concentrations after the dietary supplementation. Concentration of glucose and insulin did not change after the study period, and also, regular intake of orange juice did not induce significant changes on marker of insulin resistance (HOMA-IR) in the investigated sample. But participants of the control group presented HOMA-IR threshold higher than 2.71, which can be a risk for development of insulin resistance. Assessment of the inflammatory profile showed that the serum levels of CRP decreased by 77% in the juice group (Table 3). The regular intake of orange juice decreased TBARS (44.5%) in the juice group patients and improved the total antioxidant capacity (2.5%) in these patients while the antioxidant capacity in the control group patients decreased (1.2%) during the experimental period, without changes in the lipid peroxidation (Table 3).

**Table 3.** Biochemical and oxidative stress measurements of chronic hepatitis C patients before and after supplementation of orange juice.

	Orange juice (*n* = 23)		Control (*n* = 20)	
Parameters	Baseline	After	Baseline	After
Total cholesterol (mg dl^–1^)	142 ± 24^b^	122 ± 24^a^	136 ± 29^a^	134 ± 29^a^
LDL-C (mg dl^–1^)	74 ± 23^a^	61 ± 24^b^	79 ± 24^a^	79 ± 23^a^
HDL-C (mg dl^–1^)	41 ± 11	36 ± 10	32 ± 7	32 ± 7
Triglycerides (mg dl^–1^)	121 ± 51	111 ± 30	129 ± 56	118 ± 55
Glucose (mg dl^–1^)	94 ± 17	93 ± 12	96 ± 13	95 ± 14
Insulin (mg dl^–1^)	8.4 ± 5.0	8.1 ± 5.5	12.7 ± 6.0	12.4 ± 6.2
HOMA-IR	2.1 ± 1.5	2.1 ± 1.7	3.1 ± 1.9	2.9 ± 1.8
ALT (U l^–1^)​​​	66 ± 37	57 ± 31	69 ± 51	70 ± 51
AST (U l^–1^)	68 ± 33	58 ± 31	74 ± 43	72 ± 46
ALP (U l^–1^)	78 ± 27	70 ± 26	66 ± 18	64 ± 16
γ-GT (U l^–1^)	71 ± 57	65 ± 52	77 ± 65	76 ± 66
CRP (mg l^–1^)	2.59 ± 1.64^b^	0.44 ± 0.61^a^	0.68 ± 0.61^a^	0.44 ± 0.35^a^
ABTS (mM)	1.60 ± 0.04^a^	1.64 ± 0.03^b^	1.70 ± 0.02^d^	1.68 ± 0.02^c^
TBARS (µM)	2.72 ± 1.43^b^	1.51 ± 0.68^a^	3.14 ± 1.01^bc^	3.65 ± 1.67^c^

ALT, alanine transaminase; AST, aspartate transaminase; ALP, alkaline phosphatase; CRP, C-reactive protein; γ -GT, gamma-glutamyl transpeptidase.

Mean values ​​± SD before and after ingestion of 500 ml day^–1^ orange juice for eight weeks.

General linear model of repeated measures analysis followed by one-way and post-hoc LSD test, *p* ≤ 0.05.

Values with the same letter in a row are not significantly different, while the different letters are statistically significant.

The levels of the liver enzymes AST, ALT, ALP and γ-GT did not exhibit changes following the intervention (Table 3). A re-assessment of the liver metabolic enzymes in patients who exhibited levels above the normal threshold before the intervention found reductions in the serum levels of AST of the juice group (Table 4). No change was observed in control group.

**Table 4.** Effect of orange juice on liver enzymes of the chronic hepatitis C patients according to their hepatic levels before supplementation.

Enzyme level	Orange juice			Control		
Normal	*n*	Baseline	After	*n*	Baseline	After
ALT: 11–45 (U l^–1^)	6	27 ± 12	26 ± 13	8	22 ± 13	25 ± 14
AST: 11–39 (U l^–1^)	6	31 ± 7	32 ± 8	4	33 ± 3	27 ± 6
ALP: 27–100 (U l^–1^)	18	68 ± 18	60 ± 14	19	66 ± 18	64 ± 16
γ-GT: 7–58 (U l^–1^)	12	33 ± 12	31 ± 11	7	28 ± 7	27 ± 9
**High**						
ALT: > 45 (U l^–1^)	17	81 ± 32	66 ± 22	12	107 ± 36	106 ± 39
AST: > 39 (U l^–1^)	17	81 ± 26^b^	65 ± 22^a^	16	86 ± 42^b^	84 ± 44^b^
ALP: > 100 (U l^–1^)	5	159 ± 48	142 ± 48	1	nd	nd
γ-GT: > 58 (U l^–1^)	11	144 ± 67	110 ± 58	13	108 ± 67	107 ± 67

ALT, alanine transaminase; AST, aspartate transaminase; ALP, alkaline phosphatase; CRP, C-reactive protein; MDA, malondialdehyde; γ -GT, gamma-glutamyl transpeptidase.

nd: not detected.

Mean values ​​± SD before and after ingestion of 500 ml day^–1^ orange juice for eight weeks.

General linear model of repeated measures analysis followed by one-way and post-hoc LSD test, *p* ≤ 0.05.

Values with the same letter in a row are not significantly different, while the different letters are statistically significant.

## Discussion

The original design of this study was to verify if the habitual consumption of orange juice could lower the oxidative stress and inflammation of HCV patients. In order to prove this hypothesis, we have tested two groups of patients with HCV infection: one group drinking 500 ml of orange juice daily, and the other without drinking orange juice regularly. After regular consumption of orange juice, hepatitis C patients under antiviral therapy have had an improvement of the antioxidant capacity, lipid profile, liver inflammatory markers, and aspartate transaminase level. All of these results indicate that orange juice is a suitable food for chronic C hepatitis patients, with the advantage of lack of toxicity and high supply of dietary antioxidant compounds.

Patients undergoing pharmacological treatment frequently exhibit anorexia, weight loss, and lower quality of life because of the medication employed.[22] Regular intake of orange juice promoted an enhancement in the diet of the orange juice group patients by increasing vitamin C and folate intake. Although some authors have suggested that orange juice intake might result in weight gain due to the sugar content,[23] in our study, eight-week treatment with orange juice did not affect weight or body fat and thus represented a positive contribution to the maintenance of the patients’ nutritional status. According to previous studies, higher body fat and BMI were significantly associated with hepatic steatosis and it may contribute to growth of steatosis in patients with chronic hepatitis C.[24] Certainly, orange juice did not contribute to those deleterious effects, because it was not associated with elevation of BMI or abdominal fat, and did not caused changes in waist circumference, indicating a potential low risk for metabolic syndrome development.[25]

The harmful effects of chronic hepatitis C on patients’ nutritional status might be improved by a dietary pattern aimed to the special needs of this clinical condition. Before the intervention, protein intake of the juice group was below the levels recommended, whereas the carbohydrate intake was within the recommended range, and lipid intake was high.[26] Following the intervention with orange juice, the energy intake rose to 98.5% of the recommended values, and the macronutrient intake became appropriate. In the control group, the dietary energy, carbohydrate and protein were above the recommended levels, and lipids were at the maximum limit of the nutritional guidelines before the intervention. At the end the experimental period, there were no changes in food intake of patients in this group. Intakes of iron, vitamin C, and vitamin E were within the recommended range before and after the intervention in both groups, but calcium and folate remained low across the full period of the study. For both groups the sodium levels were rather high during the experiment period. To date, there are no recommendations available for the use of dietary supplements in this clinical condition. Nevertheless, some studies have already reported an improvement in patients infected by HCV with supplements of zinc,[13] vitamin E and C [27] among other nutrients.[28]

Consumption of 500 ml day^–1^ of orange juice increased dietary intake of vitamin C, naringin, ​​​and hesperidin.[8] These compounds might have contributed to the reduction of cholesterol and LDL-cholesterol levels in our patients who consumed orange juice. Before the intervention, the blood serum lipids of both groups were within the recommended range, agreeing with others who found appropriate levels of total cholesterol in patients with chronic hepatitis C compared with non-infected individuals.[29,30] Low levels of blood cholesterol are a common finding in patients with HCV, because intracellular cholesterol is used to viral replication.[30] Inside the hepatocytes, HCV replication requires both triacylglycerol and cholesterol for the production of virions. These demands are provide by an increase on genes activity related to lipid biosynthesis, while the low-density lipoprotein receptor activity is reduced.[31] In addition, HCV infection induces synthesis of fatty acids, which increases lipogenesis and contribute to the steatosis and the tumorigenesis.[32]

Statins have been associated with improvement of virological response, decreasing of liver fibrosis and incidence of hepatocellular carcinoma in patients with chronic hepatitis C. But, statin can also elevate liver enzymes and subsequently the hepatotoxicity, and because of this it is recommended only for patients with high cardiovascular risk and stable aminotransferases levels.[33,34] Therefore, statins are used less frequently in patients with chronic hepatitis C compared to those not infected.[35]

The bioactive compounds in orange juice, i.e. citrus flavonoids naringin and hesperidin, might reduce the availability of cholesterol to hepatic cell by inhibiting the ACAT2 enzyme and reduce the availability of triglycerides to VLDL assembly by decreasing the activity of MTP, thus decreasing the synthesis and secretion of these liver lipids. Inhibition of this important metabolic step in the production of VLDL stimulates LDL receptor expression, with a consequent reduction in serum LDL-cholesterol.[11] Studies have shown that after consumption of orange juice or isolated flavanones there was detected an increased concentration of metabolites of hesperidin and naringin, which was associated with hypolipidemic effects.[36–39] Therefore, citrus flavonoids can positively modulate lipid metabolism, decreasing the production and secretion of the HCV.[12]

Decreasing blood serum lipids after orange juice intake by healthy individuals has been associated not only with the action of flavanones but also with levels of vitamin C.[40] Actually, it was showed that vitamin C-rich foods, such as orange juice, boosted levels of vitamin C in the plasma similarly to the supplementation with higher levels of vitamin C, reaching a maximum concentration of 200 mg. Therefore, the consumption of 500 ml day^–1^ of orange juice that contains 250 mg of vitamin C seems to be in agreement with this observation.[41]

Oxidative stress is another prominent clinical feature associated with hepatitis C infection.[42] HCV produces more ROS than other hepatitis viruses and significantly influences the development of hepatic inflammation. On the other hand, the HCV adaptation to oxidative stress is key to its survival in the host.[43,44] Antiviral therapy also stimulates oxidative stress. Patients under anti-HCV therapy present elevated lipid peroxidation and reduced levels of the vitamins C, B and α-tocopherol, in addition to antioxidant hepatic enzyme activity.[7,45] However, studies suggest that antioxidants may interfere in HCV replication, to improve liver enzyme levels, to protect against liver cell damage and to render interferon antiviral therapy more effective.[44]

As observed in our study, the regular intake of orange juice enhanced the total antioxidant capacity and decreased TBARS concentration in the blood serum. These effects can be associated to the antioxidants compounds of orange juice. It was shown that hesperidin decreases pro-oxidant enzymes [46] and increases antioxidant hepatic enzymes.[47] This increase on antioxidant capacity can be strongly associated to vitamin C from orange juice. This vitamin is a natural water-soluble free radical scavenger with the ability to donate two electrons from the double bond of the 6-carbon. In this process, oxidized vitamin C generates a stable intermediate product, dehydroascorbic acid (DHA), which can be taken up by erythrocytes and reduced to vitamin C again via endogenous glutathione reductase.[48–50] A relevant role of vitamin C on plasma is to preserve α-tocopherol by recycling it by oxidation. Similar to vitamin C, α-tocopherol donates two electrons and become a less toxic product, α-tocopheroxyl. To be an effective antioxidant vitamin, this oxidized α-tocopherol must be reduced, but this process is slower than ascorbate recycling. Therefore, it is likely that α-tocopherol is recycled in the cell membrane by a mechanism that involves enzymatic ascorbate recycling via α-tocopheroxyl.[51]

Some studies also suggest using dietary antioxidants to reduce liver enzyme levels in HCV-infected individuals.[13,52] In the present study, the use of orange juice was associated with a reduction in serum levels of AST in patients who exhibited increased levels before the dietary intervention. Studies reported that the administration of hesperidin induced a significant reduction in serum AST, ALT, and ALP levels, in addition to reducing oxidative stress in rats with lipopolysaccharide (LPS)-induced hepatotoxicity, thus suggesting that this flavanone might protect against liver damage.[53] Similar results were observed after supplementation with naringenin alone [54] or associate with vitamins C and E in rats submitted to cadmium-induced hepatotoxicity.[55]

As hepatitis C virus infection causes liver inflammation,[2] CRP is a plasmatic protein released by the liver through stimulation by IL-6 and IL-1β in response to an acute inflammatory process; however, elevated CRP plasma levels (between 3 and 10 mg l^–1^) have been found in conditions of low and continuous chronic inflammation.[56] CRP levels are higher among individuals infected with HCV compared with uninfected individuals; however, CRP levels in individuals treated with antiviral therapy are lower in comparison to untreated subjects.[3] In our study, the patients of the control group already exhibited CRP normal levels (<1.0 mg l^–1^), and this level was not changed at the end of the study; however, the patients who were supplemented with orange juice showed elevated CRP levels at baseline, but this concentration was reduced significantly after the intervention with orange juice. Similar results were also obtained in a study conducted with healthy individuals,[57] in which orange juice intake was associated with reduced CRP and serum cholesterol levels. Consequently, those authors suggested including orange juice as a daily part of a healthy diet.

## Conclusion

To conclude, we suggest that orange juice intake should be encouraged in patients with chronic hepatitis C undergoing antiviral therapy. This suggestion is based on the positive effects of this ordinary food on lower levels of serum lipids, AST, CRP, oxidative stress and nutritional status preservation. It is suggested that these effects resulted from interactions between vitamin C and citrus flavonoids, concentrations of which can be influenced by processing techniques.
